# Quantitative micro-Raman analysis of micro-particles in drug delivery†

**DOI:** 10.1039/c8na00187a

**Published:** 2019-01-30

**Authors:** Daniele Di Mascolo, Alessandro Coclite, Francesco Gentile, Marco Francardi

**Affiliations:** Italian Institute of Technology 16163 Genova Italy frmaone@gmail.com; School of Earth Sciences, University of Bristol Queens Road Wills Memorial Building Bristol UK; Department of Electrical Engineering and Information Technology, University Federico II 80125 Naples Italy; GlassUp SRL via Corassori 72 41124 Modena Italy

## Abstract

Polymeric micro and nanoconstructs are emerging as promising delivery systems for therapeutics and contrast agents in microcirculation. Excellent assets associated with polymeric particulates of tunable shape, size, mechanical and chemical properties may improve the efficiency of delivery and represent the basis of personalized medicine and treatment. Nevertheless, lack of effective techniques of analysis may limit their use in biomedicine and bioengineering. In this paper, we demonstrated Raman Spectroscopy for quantitative characterization of poly lactic-*co*-glycolic acid (PLGA) micro-plate drug delivery systems. To do so, we (i) acquired bi-dimensional Raman maps of PLGA micro-plates loaded with curcumin at various times of release over multiple particles. We (ii) realized an exploratory analysis of data using the principal component analysis (PCA) technique to find hidden patterns in the data and reduce the dimensionality of the system. Then, we (iii) used an innovative univariate method of analysis of the reduced system to derive quantitative drug release profiles. High performance liquid chromatography (HPLC), the consolidated method of analysis of macro-sized systems, was used for comparison. We found that our system is as efficient as HPLC but, differently from HPLC, it enables quantitative analysis of systems at the single particle level.

## Abstract 2

Raman spectroscopy and multivariate analysis of data enable us to extract quantitative release profiles from microparticles in drug delivery.

## Graphical abstract

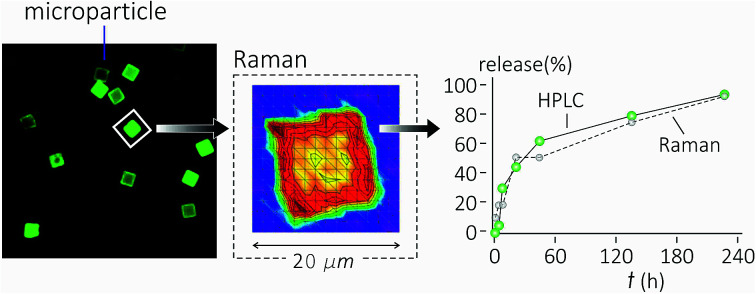

## 1 Introduction

Micro and nanoparticles have gained great interest over the last few years for use in drug delivery systems.^1–6^ Different from the conventional routes of drug delivery, micro and nanoparticles can modulate the administration of therapeutics to desired targets over time, increase the concentration of medication in the pathological districts of the body and reduce the interaction of pharmaceutical compounds with healthy tissues. Polymers are preferentially used as constituents of particles for drug release due to their versatility and efficacy. Advances in polymer science and micro-fabrication techniques have enabled the engineering of polymeric particles with precise control over their geometry, internal morphology, external surface charges and functionalization.^7,8^ Moreover, a polymeric structure may be easily modified to incorporate molecules (drug cargo) whose release will depend on specific physiological or external stimuli or the decomposition of the matrix that hosts the drug. Poly lactic-*co*-glycolic acid (PLGA) is widely accepted in the drug delivery community due to its biodegradability and biocompatibility. PLGA has been approved by the Food and Drug Administration (FDA) as a constituent of therapeutic devices.^9–11^ Precise estimation of the number of drugs or bioactive molecules trapped inside PLGA particles and quantitative particle characterization are decisive for the correct design, dosage, and modulation of pharmacokinetic properties of from delivery platforms in biomedical applications.

The current state of the art for particle characterization relies on fluorescence microscopy^12,13^ and high performance liquid chromatography (HPLC) techniques.^14,15^ Fluorescence microscopy has elevated the spatial resolution but is inherently a qualitative technique of analysis. HPLC is a quantitative analysis technique but necessitates large numbers of particles for statistical significant assessment of samples and cannot provide information of the systems at the single particle level. The acknowledgement of the limits of analysis of fluorescence microscopy and HPLC increases the need for alternative characterization techniques for micro- and nano-particles.

Raman spectroscopy is a realistic candidate for particle characterization.^16–21^ The output of Raman analysis is a spectrum in which spectral intensities are associated with the vibrational frequencies of a system.

Raman spectroscopy can be performed without pre-treatment of the samples and enables analysis in aqueous solutions or under wet conditions, that very often characterize biological samples. In contrast, Fourier transform infra-red (FTIR) systems exhibit higher performance if the measurements are carried out on dried samples, which requires pre-treatment of samples. Moreover, for their correct operation, conventional FTIR systems necessitate cryo-cooling techniques that require liquid helium or liquid nitrogen, the supply of which impairs even further sample analysis. On the other hand, high-performance liquid chromatography (HPLC) implicates the introduction of samples into a circuit along with a highly pressurized solvent, with the collateral effect of dissolving or disintegrating the samples. Raman spectroscopy differs from FTIR and HPLC in that it enables precise, non-destructive or minimally invasive sample analysis.

Raman spectroscopy is the gold standard for analyte identification, detection, and discovery. It is a precious method for estimating relative differences among substances, or to examine whether specific elements are dispersed in a solution – but insufficient reproducibility and repeatability, and the need for spectral interpretation (decoding problem) undermines Raman spectroscopy as a technique for quantitative particle characterization.

Low reproducibility and repeatability are due to several factors including the random orientation of target molecules on the substrate, temperature or humidity fluctuations, wet conditions of the measure, and electronic noise. These variables represent uncontrollable external factors during an experiment: they are all those elements that are not directly controlled by the operator and may represent a source of error in the measurements. Because of this, the intensities of the Raman spectrum of a substance express very high sensitivity to the conditions of measurements and may not be indicative of the content and chemical composition of that substance. Under standard conditions of operation, measurements of the same sample positioned differently on the stage of the Raman set-up and under the action of external environmental fluctuations may yield different Raman spectra, even if the Raman shifts of the spectra are generally preserved.

Classical Raman spectroscopy is routinely used to identify the constituents of a solution; however, for the arguments presented above, it may be inaccurate for determining the absolute content of the components of that solution.^21–27^ Raman spectroscopy is inherently a qualitative technique of analysis. Under certain conditions (liquid solution, elevated concentrations, and minimal or no interaction with the substrate), Raman spectroscopy can also be an effective method for quantitative analysis. Nevertheless, quantitative Raman spectroscopy has been mostly demonstrated in macroscopic conventional systems and solutions – it is ineffective in micro and nano-systems, and nano-medicine formulations, where its use as a quantitative quality control has to be consolidated. On the other hand, convenient post processing of data may remove external causes of error and improve the efficiency of quantitative Raman spectroscopy also in micrometric particles and systems.

More sophisticated evolutions of Raman spectroscopy, including SERS (Surface Enhanced Raman Spectroscopy), TERS (Tip Enhanced Raman Spectroscopy) and CARS (Coherent Anti-Stoke Raman Spectroscopy), are impractical for the characterization of micro particles and drug delivery systems.^28–31^

Non-confocal 2D Raman microscopy, where the working wavelength is set in the near infrared to ensure high penetration depths, may represent the optimal technique for quantitative evaluation of drug profiles and drug releases from micrometric particles. Non-confocal laser Raman microscopy is a system that irradiates a relatively low-power unfocused laser beam on the surface of the sample. In non-confocal laser Raman microscopy, the focal position of the excitation laser beam is positioned beneath the upper surface of the samples. Thus, the system enables us to analyze the internal structure of the samples with lower irradiation energy densities, still maintaining an elevated sensitivity.^32^ Moreover, the spatial resolution of non-confocal Raman microscopy may be increased using multivariate statistical methods and principal component analysis (PCA).^33,34^ In previously reported studies,^33^ multivariate analysis of Raman spectra of pharmaceutical formulations enabled significant denoising of the original data set and the generation of high quality chemical images with a higher spatial resolution and information content compared to the conventional univariate techniques of analysis. In ref. 34, Zhang and colleagues used a variety of multivariate methods to improve the resolution of chemical maps of pharmaceutical tablets, including PCA, hard and fuzzy clustering analysis methods, multivariate curve resolution (MCR), and direct classical least squares (DCLS). Using the cited methods, they segmented Raman data into spatial domains of a specific chemical composition. Thus, the final images are indicative of the spatial distribution of the chemical components in the tablets. Raman spectroscopy integrated with multivariate techniques achieves an elevated spatial resolution inaccessible to Raman analysis used in isolation. This approach has been used in the study of pharmaceutical tablets;^34^ fiber reinforced polymer composites,^35^ PLGA microsphere degradation,^36^ and tissue discrimination,^37^ in the design of functional nanostructures,^38^ and to resolve complex mixtures of proteins and other analytes.^39–41^ It is worth noting that, in all the reported cases, Raman spectroscopy has been used as a qualitative technique of analysis.

Here, we explored Raman spectroscopy as a quantitative technique of analysis of polymeric microplates (μPL) for drug delivery. Microplates are compound reservoirs for local application and delivery of therapeutics. With a size comparable with cell dimensions, *i.e.* 20 × 20 μm in length and 5 μm in height, they are visible by optical microscopy. In the present study, microplates have been loaded with curcumin, because curcumin is a trace that presents clear absorbance and fluorescence spectra. Then, they have been analysed by Raman spectroscopy at various times of release. Convenient post processing of data enabled us to extract quantitative information from the spectra and derive the quantitative profile of curcumin released by the system over time.

For analysing data (i) we have used principal component analysis (PCA) as a chemo-metric technique to reduce the dimensionality of the original data set, find patterns in the data, extract variables more representative of the system and correlate those variables to the physical characteristics of that system. (ii) We have used normalization and calibration techniques to remove possible external sources of errors from the time series.

As regarding the normalization procedure (ii): in the following of the paper we use a technique that normalizes data with respect to a standard, *i.e.* the Raman intensity of silicon measured at 520 cm^−1^. The method corrects the spectra to hold the intensity associated with the silicon constant over time and across different measurements. The intensity of remaining Raman shifts associated with the characteristics of the system is rescaled accordingly. Since the total mass of silicon is time-invariant, it is used as a standard reference respect to which all other variables are quantified. A variation of the intensity associated with a certain variable with respect to the fixed intensity of silicon indicates the percentage variation of the content of that variable over time. If the initial conditions are known, *i.e.* the exact volume of the drug loaded into the system at the initial time, the method enables us to estimate quantitatively the time evolution of that drug and its content in the system in absolute values. In essence, the dynamic normalization used in the present study is a form of calibration used to (a) remove external factors, fluctuations and external sources of error from the system, (b) adjust the output of the system and (c) allow the comparison between measurements across different times.

Results were validated with HPLC analysis of samples. The described approach, in which Raman characterization is coupled with statistical analysis of data, represents the first reported example of quantitative Raman characterization of micro-particles and drug release at the single particle level.

## 2 Results and discussion

### 2.1 Micro-plate preparation

**Fig. 1:**
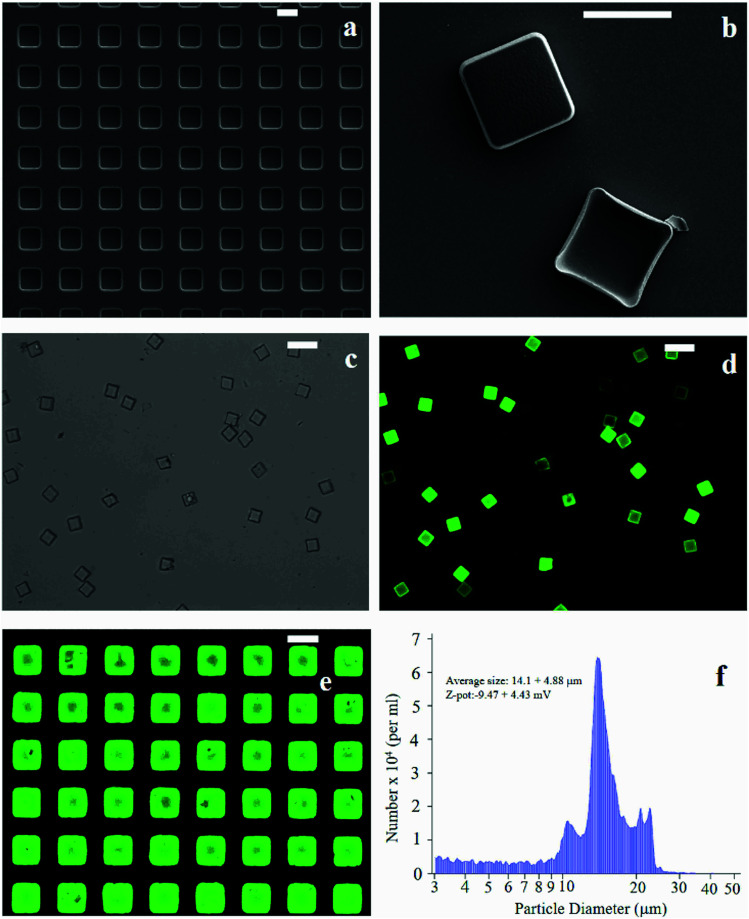
SEM micrograph of the silicon template for the fabrication of micro-plates (a). PLGA micro-plates imaged as released from the template, the upper surface exposed to air during the fabrication process exhibits a depression (b). Both in (a) and (b) the scale bar is set to 20 μm. PLGA plates loaded with curcumin are imaged through conventional optical (c) and fluorescence microscopy where particles appear in bright green (d). Both in (c) and (d) the scale bar is set to 50 μm. In (e), a PVA template with a curcumin-loaded μPL before release is shown (the scale bar is set to 20 μm). Multisizer analysis performed on μPLs after release is presented in (f).

The fabrication of micro-plates starts with the production of a silicon master template. A SEM micrograph of the template shows square wells (that are, the plate negatives) distributed in ordered arrays over large areas of a silicon substrate (Fig. 1a). This assesses the fabrication process capability to attain maximum control over the shape and size of the microplates and secure reproducibility and repeatability. SEM (Fig. 1b), optical (Fig. 1c) and fluorescence microscopy (Fig. 1d) images of the polymeric microplates released from the template confirm the morphology of individual particles that are cuboids with a base length of 20 μm and a height of 5 μm. The particle shape and particle size are regular within the imaged regions with small or negligible deviations from the mean. An individual silicon template may release up to ∼10^9^ particles in a high-throughput and efficient fabrication process where flawed or waste particles are a small percentage of the total. The moderate depression over the upper surface of the plates as shown in Fig. 1b may be ascribed to the rapid chloroform evaporation that occurs from the surface when exposed to air (Methods). The fluorescence image of curcumin loaded within the plates (Fig. 1d) shows that the payload is uniformly distributed inside the systems. This is also shown in Fig. 1e where the fluorescence characterization of PVA molds filled with the PLGA + curcumin paste is presented. μPL bunches are characterized using a multisizer tool, extracting an average size of 14.1 ± 4.88 μm and a *Z*-pot of −9.47 ± 4.43 mV. These data are reported in Fig. 1f. Note that for this configuration, the overall dimension of the plates is large and is not compatible with the transport in the microcirculation. The described plates are not intended for injectable use but for site-specific and local delivery of therapeutics.

### 2.2 Raman analysis and PCA data reduction

**Fig. 2:**
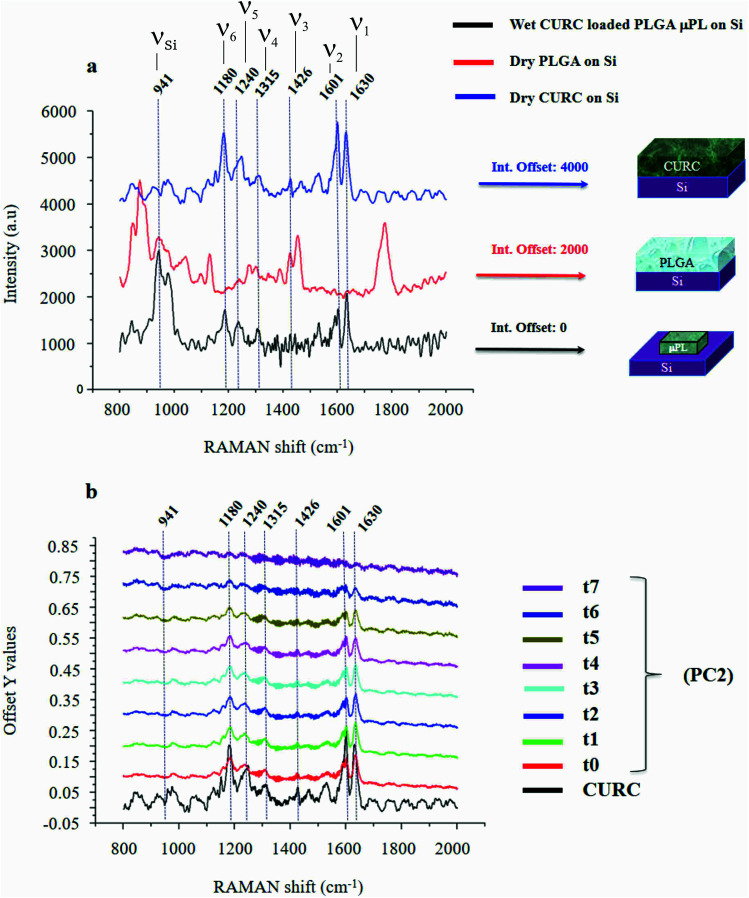
PLGA micro-plates were characterized using micro-Raman analysis. Measurement and cross comparison between the Raman spectra of bulk curcumin (blue), bulk PLGA (red) and the PLGA platelet (black) allow us to define the Raman profile of the system (a). Raman spectra of micro-platelets loaded with curcumin at different times of release, the information content of the entire system is delivered by the sole *ν*_1_ frequency (b).

Fig. 2a shows the Raman signature of a (i) PLGA microplate loaded with curcumin compared to the signal of (ii) bulk curcumin and (iii) bulk PLGA. For measuring the samples, the spectral range was set between 800–2000 cm^−1^ to display all acquired spectra in the same limits. Moreover, experimental conditions were maintained fixed for all the acquisitions as described in the methods. The microplate spectrum (i) is characterized by seven Raman shifts and these are labelled in Table 1. Six of these modes may be attributed to curcumin (*ν*_1_–*ν*_6_); one mode is instead related to the silicon substrate *ν*_Si_ = 941 cm^−1^.^42^ Moreover, spectral intensities of the microplate (i) associated with frequencies greater than 1650 cm^−1^ are vanishingly small and the spectrum itself is mostly flat, this would indicate that curcumin in the plate is in the form of enol (Table 1). The acquisition time for bulk PLGA was longer than that required for analysing pure curcumin and microplates due to its amorphous structure. On comparing the signals of the (i) microplate and (iii) bulk PLGA, it can be noticed that the characteristic Raman peaks of PLGA are not present in the spectrum of the microplate. This is easily explained considering that, at the micro scale, the amorphous structure of PLGA is transparent to visible radiation.^43^ We also observe that the Raman intensity measured at the Raman shift *ν*_3_ is negligible in the Raman spectrum of the micro-plate: for this reason, it is neglected in the rest of the analysis.

**Table 1:** Principal Raman modes of curcumin measured in a micro-plate extracted from the spectrum in Fig. 2. In the inset, we report the wiring diagram of the molecular structure of curcumin

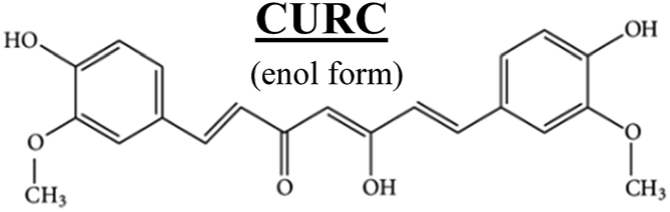	Raman shift@*λ*_ex_ = 785 nm cm^−1^	Peaks label (*ν*) vibrational–(*δ*) rotational
*ν*_1_	1630	(*ν*) carbonyl C=O
*ν*_2_	1601	(*ν*) aromatic C=C
*ν*_3_	1426	(*ν*) phenol C–O
*ν*_4_	1315	(*δ*) phenol CCHCOH enol
*ν*_5_	1240	(*ν*) enol COH
*ν*_6_	1180	(*δ*) CH_3_
*ν*_Si_	941	(*ν*) Si (second-order)

We used PCA to analyse data and reduce the dimensionality of the original data set (Methods). Upon convenient post-processing of data we described the Raman spectra of the system through three sole principal components: PC1, PC2 and PC3. In what follows, using arguments based on the physical observation of the system, we demonstrate that the first principal component PC1 accounts for silicon, the second principal component PC2 encodes information about curcumin, while PC3 cannot be correlated with sufficient confidence to none of the variables of the system, including PLGA (ESI Fig. S1.1†).

**Fig. 3:**
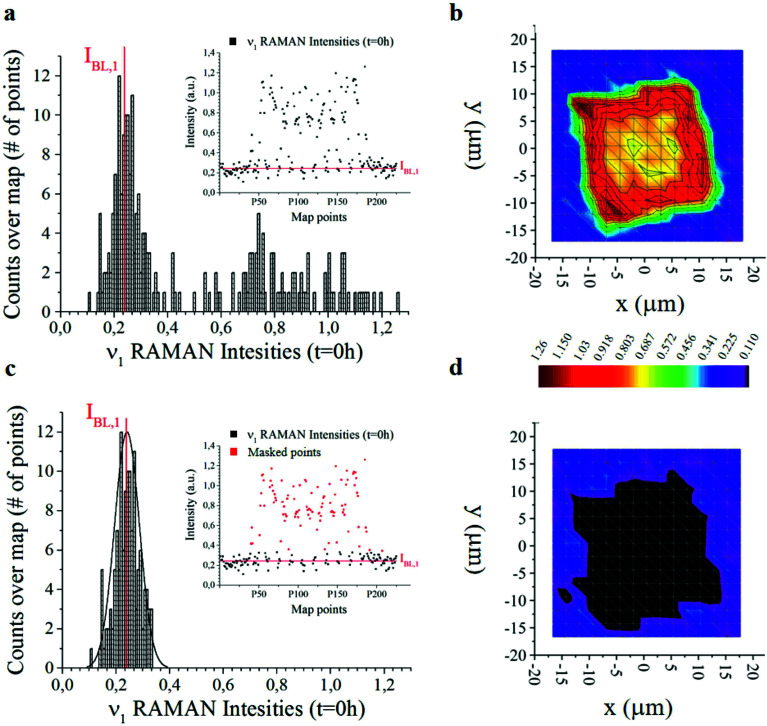
Histogram of 255 Raman intensities associated with the *ν*_1_ frequency measured over a micro-plate at the initial time of release *t* = 0. The intensity with the highest number of events is the baseline *I*_BL,*j*_ here marked in red. Notice that *I*_BL,*j*_ ≠ 0 (a). The distribution is used to construct bi-dimensional intensity maps of the micro-plate (b). Same graphical representation of data as in (a and b) with a set of data-points (marked in red) removed from the analysis (c and d). From the comparison of the contour plots (b) and (d), one can deduce that the points that contribute to the Gaussian peak in the histograms are positioned outside the particle and represent the silicon background.

The analysis was performed over 225 Raman spectra for a particle, with 9 technical repeats for the particle, and 1 experimental repeat for each time set in the 0–240 h interval. For each technical repeat, Raman spectra were acquired using a region of interest larger than particle size (Fig. 3) – thus the spectra contain either relevant information about the system or the background. Since the background is constituted mostly by silicon, that is an invariant of the system, it may be used as a reference to normalize data and calibrate the entire system. This enables us to extract otherwise inaccessible information from the system. The following is dedicated to the articulation of this thesis.

The physical system under examination has three constituent elements: the (i) silicon substrate, the (ii) curcumin contained in the particles, and the (iii) PLGA polymer forming the particles. Upon application of PCA to the original data set, we found that the first 3 principal components explain 98% of the total variance of the system. This justifies the decision to limit the analysis to the first three components. Coincidentally, the number of relevant components resulting from the PCA analysis coincides with the number of elements of the system. Loadings associated with the first three components are reported in ESI Fig. S1.1 and 1.2.† From the plots, we can observe that the loading profile associated with the first component (PC1) matches with the Raman spectrum of silicon, the loading associated with the second component (PC2) correlates to the Raman spectrum of curcumin, while the loading associated with the third component (PC3) is not coherent with the Raman profile of PLGA or silicon. We can conclude from observations that the first principal component is representative of silicon, while the second principal component is representative of curcumin. We calculated a chi squared statistics *χ*^2^ to test whether the PC2 loading profiles match with the Raman signature of curcumin. Values of *χ*^2^ near unity indicate that the signal of curcumin is consistent with the matching template. Thus, we can use the PC2 variables to examine the behavior of the system and determine its release dynamics. The first principal component PC1 is the component that retains the majority of the variability of the system. The observation that most of the variability of the system may be ascribed to silicon is justified as follows. The PC1 signal, associated with the silicon substrate, is acquired on and mediated by a large area that comprises the particle, the curcumin contained in the particle and the silicon background. As the curcumin in the particle is released and removed from the system, the relative content of silicon in the whole dataset varies. On the other hand, since curcumin is evenly distributed in the particle, its variation is less intense than that correlated to silicon. This is reflected by a first principal component correlated to silicon and a second principal component correlated to curcumin. The contribution of other components to the analysis is negligible. In a configuration where the region of interest (ROI) overlaps with the area defined by the particle, most likely we would observe an inversion between the first and the second principal components, *i.e.* PC1 would be associated with curcumin and PC2 to silicon.

The correspondence between the second principal component of the system and the Raman profile of curcumin is verified each time of the analysis. Loading profiles associated with the second principal component PC2 are reported in Fig. 2b for various times of release ranging from *t*_0_ = 0 h to *t*_7_ = 240 h; the Raman spectrum of bulk curcumin is reported in the same diagram for comparison. The loading curve associated with a principal component is a function that indicates which frequencies have the highest variability and therefore carry more information. This describes the contribution of each frequency to a specific principal component. In Fig. 2b, one can observe the emergence within the loading profile associated with PC2 of several Raman bands, where the shifts of the bands exhibit a tight correspondence to the shifts of the principal peaks of the spectrum of curcumin, while the intensities decrease over time. Since we found that the totality of Raman shifts of the peaks in the PC2 loading profile match with those of curcumin with a 100% confidence, in the rest of the paper we assume that PC2 encodes the content of curcumin in the microplates at each time of the analysis. For the same reasons, the signal coming from the silicon substrate can be attributed to the first principal component PC1.

In ESI Fig. S1.1a,† the first principal component loading curves PC1, PC2, and PC3 are reported for different particles at the initial time *t* = 0 for the sake of clarity. Profiles associated with PC1 and PC2 are consistent and are identical across different particles. Loading profiles associated with PC3 are instead uncorrelated and present peaks (valleys) randomly oriented upward (downward) moving from a sample to another sample.

### 2.3 Identification of information carriers of the system

It is observed that (i) the intensities associated with the frequency bands *ν*_4_, *ν*_5_ and *ν*_6_ in the PC2 loading profile are less intense compared to the intensity associated with *ν*_1_, and that (ii) mode *ν*_2_ is the convolution of two pure state frequencies (the mode results from the convolution of modes associated with the fundamental forms of curcumin, *i.e.* keto form and enol form) and thus cannot be considered as a carrier of information (Fig. 2b), so the content of curcumin in the microplates can be related to in the sole *ν*_1_.

PCA is akin to a roadmap intended to guide the choice of the most relevant parameters of a complex system but is not meant for the quantitative analysis of that system. Here, PCA was used to identify the most informative frequency of the system (*ν*_1_), additional data analysis, based on a type of data normalization, and enabled quantitative characterization of the dynamics of drug release.

On reporting the first two principal components PC1 and PC2 in a scatter plot (ESI Fig. S1.2c†), one can observe that the components are orthogonal, *i.e.*, variation associated with silicon is linearly independent from the variation associated with curcumin. Thus, silicon and curcumin are uncorrelated, and this justifies the use of the silicon Raman intensity for normalizing the data. A Raman spectrum of pure silicon is shown in ESI Fig. S2.†

### 2.4 Post-processing of Raman data

The entire post processing of data is a two-step procedure (Methods).

(1) At the first stage, all spectra are normalized with respect to silicon, which is a physical characteristic of the system. Normalization is, in turn, performed in two phases. We first correct the spectra with respect to the intensity measured at *ν*_Si/1_ = 520 cm^−1^ – that is assumed not to vary over time (the normalizing constants are reported separately in ESI 2†). Then, we normalize spectra with respect to the phononic peak of silicon measured at *ν*_Si/2_ = 941 cm^−1^. The first classical normalization allows the comparison of data across different times. The second normalization enables the comparison between data within the same group.

(2) At the second stage, distribution of Raman intensities as that reported – for example – in Fig. 3 and physical observation of data, allows one to segregate the signal coming from the background (*i.e.* noise, N) from the signal coming from the particle. Then, we subtract the noise from the overall signal, to obtain an estimate of the maximum available information derivable from the system, *I* = S − N.

Normalization with respect to the silicon substrate (1) correlates data to a standard reference, thus enabling quantitative analysis.

Subtraction of the background – resulting in an offset of the data set – (2) removes systematic noise enhancing efficiency, increasing precision and assuring reliability.

The offset of data is based on the analysis of the distribution of Raman intensities measured on a particle (255 points) for the *ν* = *ν*_1_ frequency associated with the curcumin content in the system. Raman intensity events at the initial time *t* = 0 h show a Gaussian profile (Fig. 3a). The average intensity of this distribution *I*_BL,*j*_ is the non-zero signal of curcumin coming from the silicon substrate. It represents the baseline against which all Raman intensities in the map are positioned, for a specific frequency *ν* = *ν*_1_.

*I*_BL,*j*_ is the average value of the intensities of the spectra associated with *ν*_1_, *i.e.* the main carrier of information of curcumin. As such, we expect that its value is zero when the signal comes from the silicon substrate. Under realistic conditions – because of external sources of errors, electronic noise, environmental noise, the signal coming from the background, imperfect adhesion of the particle to the substrate and related perturbations – *I*_BL,*j*_ is different from zero. Thus, *I*_BL,*j*_ is a comprehensive measure of the systematic error affecting Raman acquisition. As such, it can be compensated.

We therefore offset all the intensities in the distribution by subtracting *I*_BL,*j*_ – Fig. 3c highlights the signal coming from the sole silicon substrate. In doing so, the signal of curcumin coming from the substrate and outside the particle is set to zero making possible quantitative comparisons across measurements. This operation is akin to polynomial baseline corrections in conventional Raman software and applications, but in our method the baseline position is determined from the physical observation of the system and the silicon background and is not limited to the mathematical manipulation of data. Different from standard methods of baseline correction, data are compared to a signal with a physical significance, *i.e.* the silicon background. Since the content of silicon in the system is constant, this guarantees the reliability of the results.

Another relevant difference is that, in our method, there is no need to baseline correct data –over the entire spectral range. Once the signal associated with the background (*i.e. I*_BL,*j*_) is determined, to gain information about the system, it is sufficient to correct the intensity associated with the sole frequencies relative to the curcumin content, *i.e.* frequencies *ν*_1_ to *ν*_6_, and especially frequency *ν*_1_. The output of the whole process of data analysis is not a spectrum, it is a finite set of corrected intensities correlated to very few Raman shifts, at most six. The evolution of intensities over time gives quantitative information about the release mechanisms of the drug delivery system, as explained in detail in the following paragraphs 2.5 and 2.6. Assuming as a standard reference the physical characteristic of the system (*I*_BL,*j*_) and relating all data to that standard, is a key determinant for deriving quantitative release profiles.

In Fig. 3b and d we report the 2D distribution of Raman intensities before and after background subtraction. Note though that the effect of the background correction is not appreciable until comparison with HPLC results. For the present study, 9 samples were analysed at different time points, namely 0, 6, 10, 24, 48, 144 and 244 h. 225 Raman spectra were acquired for each sample over different points of a map and post-processed as described above.

### 2.5 2D density maps of curcumin

**Fig. 4:**
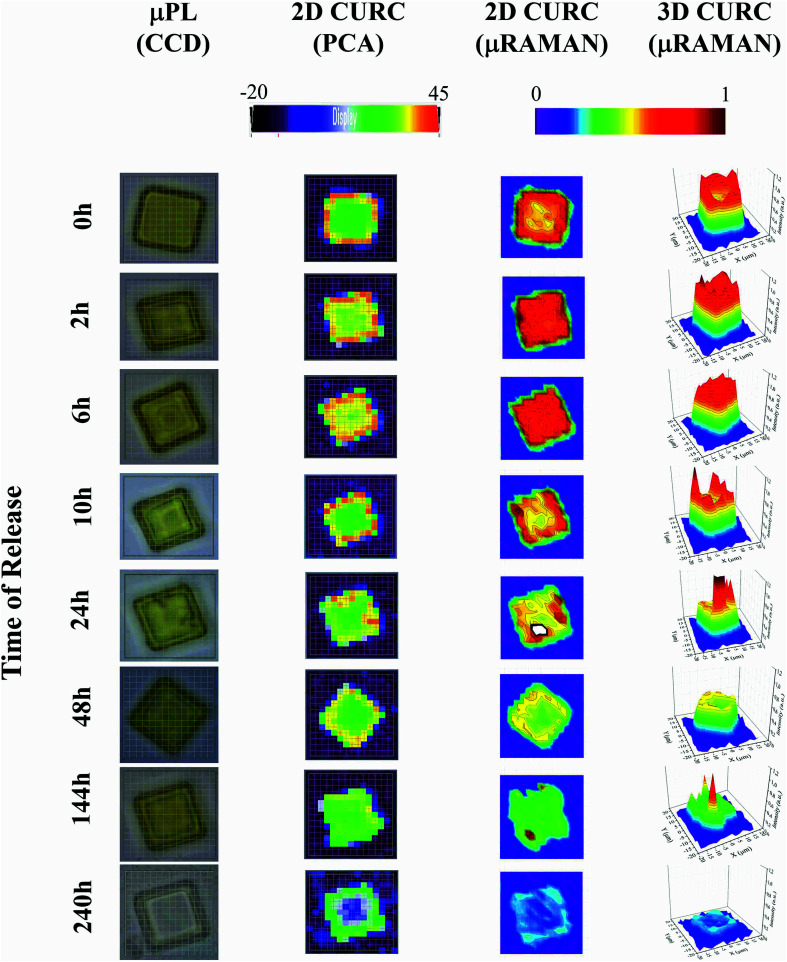
The figure displays the micro-plate at different times of release. From left to right: optical image of the particles acquired through a CCD camera integrated in the Raman set-up (first column). Distribution of curcumin in the plate reconstructed using the second principal component PC2 of a principal component analysis carried out using the WiRE software (second column). Bi- and three-dimensional curcumin profile obtained by a convenient post-processing of Raman spectra for a fixed *ν*_1_ frequency (third and fourth columns).

The method was used to build quantitative 2D and 3D curcumin intensity maps at different times of release (third and fourth column in Fig. 4), with a higher detail and a better resolution compared to curcumin distribution profiles reconstructed by simple PCA (second column in Fig. 4) or optical microscope imaging (first column in Fig. 4). An optical inspection of the system (left column in Fig. 4) shows no or minimal degradation of the PLGA up to 240 h from release, suggesting that the delivery mechanism is based on pure diffusion rather than on the erosion of the polymeric matrix and gradual release of the therapeutics trapped inside. 2D and 3D maps in Fig. 4 describe the evolution of the system over time and the mechanisms of release of curcumin from the plates. Assuming that drug delivery is mediated by diffusion and that the fluid in which plates are immersed is at rest, the concentration of curcumin over time is described by the first law of Fick^44,45^

1 *J* = −*D*∇*c*

where *J* is the molar flux of molecules, *D* is the Brownian coefficient of diffusion, and ∇*c* is the spatial gradient of concentration. Thus, curcumin molecules are preferentially transported in the domain from higher to lower density regions. At the initial time of release (*t* = 0), curcumin is concentrated in the periphery of the particle. Uneven distribution generates radial molar fluxes from the border (high concentration) to both (a) the external surface of the particle (where the concentration *c*_a_ is vanishingly small) and (b) the internal region of the particle (where concentration *c*_b_ is low but not zero). Considering that *c*_a_ < *c*_b_, a positive net flux of curcumin is directed to the outside of the particle, removing the substance from the system and reshaping the concentration profile of curcumin from a doughnut-like shape to a uniform distribution. After 6 h from release, curcumin is evenly distributed within the microplate. The imbalance of concentration between the microplate and the external environment generates again fluxes of the analyte and a gradual consumption of curcumin that is completely removed from the system after 240 h from release. For a similar particle configuration, drug delivery is sustained over time for a maximum of 10 days. The results presented in Fig. 4 and interpretation thereof may not be obtained by applying a classic Raman post-processing and the presented results go beyond the current state of the art Raman or other conventional techniques of analysis. Due to an elevated penetration depth of the wavelength *λ*_ex_ = 785 nm of the laser radiation chosen for the analysis, curcumin concentration profiles were derived within the entire volume of the microplate and are not limited to its superficial layer.

### 2.6 Comparison with HPLC

**Fig. 5:**
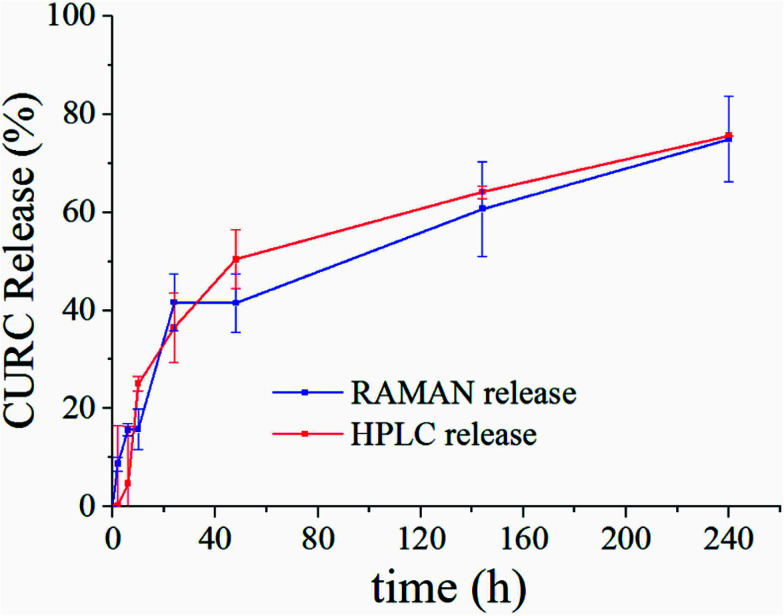
Curcumin release curve reconstructed by our proposed method of analysis of Raman spectra (blue) compared to HPLC (red). Traces are obtained by independent unsupervised experiments on the same sample. The good matching between release curves demonstrates the correctness of the method.

Fig. 5 shows the release profile of the microplate system obtained with our method compared to its characterization by HPLC. Traces in Fig. 5 describe the variation of the curcumin content in the system expressed as a percentage with respect to the initial time of release. Remarkably, Raman and HPLC release curves are nearly coincidental with deviations from the mean that lie well within error limits. This confirms the correctness of the proposed Raman approach and data analysis. The main difference between HPLC and Raman spectroscopy lies in the way the analysis is carried out. While HPLC is performed on the entire population of particles, Raman spectroscopy is conducted at the single particle level. For comparison, HPLC measurements were performed on a set of particles purified of PLGA debris or other fragments, which may significantly influence the output of the analysis. In ESI Fig. S4.1† we report the HPLC release profile of curcumin derived from an untreated set of micro-particles. We may observe that (i) the release is artificially augmented at all the times of the measurement, and (ii) at the early time of release, delivery is accelerated (we observe a boost in the time profile) due to the rapid diffusive dynamics of small debris compared to larger micro-particles. Thus, the described Raman quantification method may be considered a standard to assess the purity of samples in that, different from all other reported methods of analysis, it is not affected by impurities or sample imperfections.

Furthermore, once a preliminary PCA analysis isolates the frequencies that describe the system, one can perform further Raman analysis in narrow spectral intervals, centered around the pivotal frequencies of the system, assuring a higher spectral resolution. Scanning wider regions of the spectra with the same high resolution would require performing the acquisition repeatedly moving the acquisition window over the entire interval – that is time consuming and lengthens the time of the analysis. Thus, focusing on a limited number of frequencies indirectly reduces the acquisition time.

The test campaign that we presented in the paper was not focused on optimizing performance and was more an exercise proof of existence. While a single particle cannot be representative of a large sample of particles, it enables us to examine in detail the constituent elements of a drug delivery system, extract information from that system, and record its evolution over time. Different from HPLC, that is performed on a sample of many elements without distinction between elements, our method resolves individual particles in a sample. Moreover, our method can be potentially applied to a smaller number of particles extracted from a more numerous population to determine whether it meets a predetermined standard of quality in a statistical approach. The increased information quality, quantity, and density associated with our method can provide valuable support to those working in the field of drug delivery, biomedical engineering, and bio-nanotechnology.

A comparison of our method with alternative mathematical techniques of analysis is reported separately in ESI 5 and 6.†

## 3 Conclusions

We demonstrated Raman Spectroscopy for the quantitative, single particle-based analysis of drug delivery in PLGA micro-sized plates. The release profile of curcumin in individual particles was derived over time up to 240 h and compared to HPLC analysis, which is the consolidated standard for analytical quantification and analysis. The fraction of released curcumin estimated with our method matches with the measured HPLC profile with a satisfactory level of accuracy for all the times of analysis. Nevertheless, while HPLC necessitates large numbers of particles for the sample assessment, the proposed method is effective at the single particle level and extends the spatial resolution of current techniques of analysis. In our approach, sample characterization and quantification is enabled by convenient post processing of Raman spectra. While principal component analysis (PCA) finds hidden patterns in the data and reduces the dimensionality of the system, a normalization technique removes noise from the data and eliminates external sources of errors. Thus, the combination of statistical techniques of analysis with Raman Spectroscopy allows us to derive quantitative information from micro-sized systems for applications in drug delivery, pharmacology, bio-nanotechnology, health, safety and environment.

## 4 Materials and methods

### 4.1 Synthesis of microplates

Microplates (μPL) are PLGA cuboids with a base length of 20 μm and a height of 5 μm (Fig. 1). Samples were obtained from multiple replica-molding steps, starting from a silicon master template, as described by Di Francesco *et al.*^46^ Briefly, the surface of a silicon wafer is patterned with an array of rectangular wells of the aforementioned dimensions, separated by a gap of 20 μm, extended over large areas, up to 3 × 3 cm (Fig. 1a). The master template was micro-fabricated using optical lithography and deep reactive ion etching techniques.^10^ Upon fabrication, it was first replicated into PDMS templates, and then into PVA water soluble polymer duplicates, exactly reproducing the silicon one. This double process is necessary to obtain multiple sacrificial templates, as the PVA is easily dissolved in water, conversely to PLGA, hence allowing the simple separation of the single μPLs from the template. Moreover, from one initial silicon template, it is possible to obtain numerous templates. PVA molds were filled with a paste of 5 mg of PLGA and 500 μg of curcumin in acetonitrile for a 3 × 3 cm template. Upon evaporation of the solvent, the loaded PVA was dissolved in DI water (Milli-Q), thus releasing curcumin-loaded μPLs (curcumin-μPLs).^8^ Microplates were finally purified using serial centrifugations, the first at 1000 rpm for 5 minutes and the second at 1000 rpm for 2 minutes. μPLs obtained at the end of the 2 centrifugations can be considered highly purified compared to μPLs obtained after only the first one, as small polymeric debris still present in the solution, including fragments of damaged curcumin-μPLs, can be more easily pelleted under the conditions of the first centrifuge.

### 4.2 Release study

Microplates were suspended in PBS and 200 μl of this solution was placed in dialysis cups (3 per each time point), immersed in 4 l of PBS at 37 °C. The curcumin release profile was obtained at different time points, namely 2, 6, 10, 24, 48, 144, and 240 h, using independent Raman and HPLC analysis. For Raman characterization, 5 μl of the cup solution were withdrawn and spotted on a silicon support. Upon evaporation of the solvent, particles were rehydrated using DI water (Milli-Q) to ensure adhesion of the μPLs to the silicon substrate, previously cut with precision to maintain a fixed orientation of their axis; this enables the data normalization for drug quantification, as described later. During acquisition, temperature of the system was maintained fixed at 21 °C. By measuring the particles, it was found that the integration time was negligible compared to the total time of the release. For HPLC, a 1260 Infinity HPLC Agilent was used. 3 samples per time point were collected, centrifuged (1000 rpm for 5 min) and re-suspended in a 1 : 1 solution of acetonitrile and water to dissolve particles. The curcumin content was obtained by using its absorbance peak at 430 nm, under isocratic conditions, as per acetonitrile : water, and estimated by interpolation with a standard curve.

### 4.3 Micro-Raman set-up

An inVia Micro-Raman set-up from Renishaw, equipped with a *λ* = 785 nm CW laser source, was used for acquiring Raman spectra. An Andor-camera technology detector, with a 1200 lines per mm grating, a spectral resolution of 0.1 cm^−1^, laser power *P* = 200 μW, a 60× WI and 50× objectives were used for all the measurements. In the experiments, the optical source was linearly polarized.

### 4.4 Software support

The commercial software WiRE 3.4 by Renishaw was used for set-up managing, data acquisition and analysis (spectra post-processing and PCA). Remaining statistical analysis and the generation of curcumin bi-dimensional maps were performed in OriginPro 9.1.0 by OriginLab.

### 4.5 Acquiring Raman spectra

The laser was switched on 20 minutes before the sample measurement. Raman spectra of p-doped, 100, 10–20 ohm cm^−1^, silicon substrates were acquired as a reference and to correct fluctuations of the power source over time. The following settings were used for acquiring the reference silicon spectra and sample spectra: laser power *P* = 10 mW and exposure time *t* = 0.1 s. For silicon spectra, a 50× objective in air and a frequency spectral range of 100–3200 cm^−1^ were used. The intensity of the spectrum of silicon measured at 521 cm^−1^ was used for the first normalization of data at different time points. For acquiring sample spectra, we used the following procedure: (i) each particle was centered in a squared region of interest (ROI) with 37.5 μm edge length; (ii) Raman spectra were acquired with a resolution of 2.5 μm in both directions resulting in 225 total spectra per sample; a (iii) 60× WI objective was used for measurements in a wet environment; (iv) 800–2000 cm^−1^ spectral range. These settings avoid PLGA degradation, still assuring an elevated spectral resolution. It must be noted that the orientation of the particles in the ROI is random. For bulk curcumin and bulk PLGA the acquisition was done under dry conditions.

### 4.6 Post-processing of data

Post-processing of the data combines PCA multivariate analysis as a first step and univariate analysis as a second step, according the following procedures:

(i) Row data for each sample were processed using the PCA module installed in WiRE 3.4. Then, the profiles of curcumin loaded inside μPL profiles (from now on “loading profiles”) and scatter plots (ESI Fig. S1.1 and 1.2c,† respectively) associated with the first three principal components PC1, PC2 and PC3 were derived.

(ii) The PC2 loading profile, that provides information about the curcumin content in a particle, is super-imposed on the Raman spectrum of bulk curcumin. In this way, 6 characteristic modes (*ν*_*j*_, *j* = 1, …, 6) are correctly assigned to curcumin (Fig. 2 and Table 1). For particle analysis, the sole *ν*_1_ = 1630 cm^−1^ Raman shift was used, because it retains the higher level of information. The peak measured at *ν*_Si_ = 941 cm^−1^ within the PC1 loading profile may be ascribed to the phononic resonance of silicon in the second order and was used for the second normalization procedure.

(iii) Data smoothing was performed using the Savitsky–Golay algorithm built-in WiRE through convolution with a polynomial function of the 3^rd^ degree over an interval of 25 sample points.

(iv) Silicon Raman spectra were elaborated using Origin. A 500.7–545.4 cm^−1^ spectral region centered on the principal band of silicon at *ν*_Si_ = 520.3 cm^−1^ is isolated and the peak is fitted using a Voigt function. The intensity of this peak was considered equal to 1 for each time point.

(v) Raman data acquired per each particle are organized in a *A*_255,7_ matrix where the *p* = 255 rows of *A* contain the relative intensities *I*_*i*,*j*_ and *I*_*i*,Si_ per each of the 255 points of the map reported for the characteristic frequencies of curcumin (6, *I*_*i*,*j*_) plus the characteristic intensity of silicon (1, *I*_*i*,Si_), for a total of 7 columns.

(vi) A second normalization is performed for each spectrum whereby *I*^N^_*i*,*j*_ = *I*_*i*,*j*_/*I*_*i*,Si_.

(vii) Observation: the silicon intensity measured at 520 cm^−1^is an independent recording performed before effective particle analysis. As such, it is used as a reference for the calibration of the entire system. Notice though that the Raman scan of (1) silicon and (2) the particle is carried out under the same experimental conditions –silicon itself is the sample holder and is not removed or displaced across different measurements.

(viii) Distribution of *I*^N^_*i*,*j*_ Raman intensities is reported in a histogram for all the points in a map for each of the characteristic frequencies of curcumin *ν*_*j*_ (Fig. 3 and ESI Fig. S3.1†). Points in the histogram following a Gaussian distribution may be associated with the measurements outside the particle. By changing *ν*_*j*_, the origin of the Gaussian distribution varies. This can be considered as a baseline value, *I*_BL,*j*_, that is subtracted from the original intensities to obtain the final, correctly offset values of Raman intensities *I*^N,BL^_*i*,*j*_ = *I*^N^_*i*,*j*_ − *I*_BL,*j*_.

(ix) For each particle, *I*^N,BL^_*i*_ intensities are plotted as a function of the spatial *x*, *y* coordinates for specific *ν*_*j*_ modes. Thus, a contour-plot and a 3D plot, in which per each point *x*, *y* of the particle, the *z* coordinate of the plot represents the Raman curcumin intensity measured in that point, were obtained (Fig. 5).

(x) Quantitative statistics permits to derive, per each particle, the sum of curcumin Raman intensities 
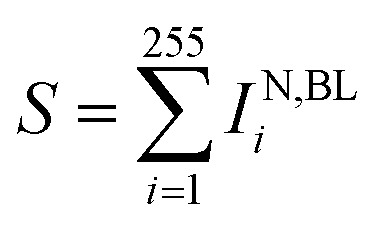
 for specific *ν*_*j*_ and times of the measurement.

(xi) Per each time point, *S* is averaged over all the *n* particles analyzed, here *n* = 9. The average value of *S* is expressed as the percentage with respect to the initial time of release *t* = 0 h. Points in the curve are calculated as the Raman intensity measured at *ν*_1_ to the Raman intensity measured at *t* = 0 h. Standard deviations are influenced by three factors: (i) number of analysed micro-platelets per time-point; (ii) ratio of the total volume of the particle to the volume analysed by Raman spectroscopy; (iii) variability of the Raman signal measured within the same particle. In the presented experiment, the parameters of the process, including the number of particles, step-size, and integration time, are chosen to yield maximum accuracy in drug quantification.

## Data availability

The datasets generated during and/or analysed during the current study are available from the corresponding author on request.

## Author contributions

MF led the entire project and wrote the paper. He developed the innovative analytical Raman post-processing, acquired the Raman spectra and micro-fabricated the silicon template for micro-particles. DDM has synthesized the particles loaded with the curcumin and performed the classical characterization on them (*Z* pot-Multisize-HPLC). He has contributed to numerous and fundamental scientific discussions. AC contributed by scripting MatLab codes to automate the post-processing of the numerous Raman spectra for each particle. FG performed multivariate analyses (PCA) and contributed to numerous scientific discussions needed to achieve the presented results. He contributed to the writing of the manuscript.

## Conflicts of interest

The authors declare no conflicts of interest.

## Supplementary Material

NA-001-C8NA00187A-s001
