# Supplementary material for: Quantitative micro-Raman analysis of micro-particles in drug delivery
Source: Nanoscale Adv. 2019 Jan 30;1(4):1541–52. doi: 10.1039/c8na00187a (PMC6592161; doi:10.1039/c8na00187a)
Supplement: NA-001-C8NA00187A-s001 [file NA-001-C8NA00187A-s001.pdf]

Electronic Supplementary Material (ESI) for Nanoscale Advances.
This journal is © The Royal Society of Chemistry 2019

Quantitative micro-Raman analysis of micro-particles in drug delivery

Daniele Di Mascolo[1], Alessandro Coclite[2], Francesco Gentile[3], Marco Francardi[1,4*]

[1]Italian Institute of Technology, 16163 Genova, Italy

[2]School of Earth Sciences, University of Bristol, Queens Road Wills Memorial Building, Bristol UK

[3]Department of Electrical Engineering and Information Technology, University Federico II, 80125 Naples, Italy

[4]GlassUp SRL, via corassori 72, 41124, Modena, Italy

***** author to whom correspondence should be addressed**:** frmaone@gmail.com

Supporting Information

SI-1. Principal Component Analysis applied to 2D μRAMAN mapping. The application of chemiometry to spectroscopic data interpretation by multivariate analysis (i.e. Principal Component Analysis, PCA) is well known. PCA is an orthonormal linear transformation of a set of variables (i.e. intensity at specific frequencies in spectrum) in a set of uncorrelated functions of the same variables called Principal Components (PCs). The applied transformation is based on the covariance matrix (**S**) of the measured variables in a way that the PCs are listed in function of the grade of variance of them, from the higher to the lower. The application of PCA to μRAMAN map highlights that each function is ascribable as the spectral fingerprint of a chemical element that varies independently with respect to other. The variables of these functions are the shifted RAMAN frequencies to which is assigned a *score* as a function of their variability. Therefore to each chemical element is possible to match a PC. As described in Materials and Methods section, the pre-installed module on WiRE performs the PCA on each μRAMAN map. In the **Supporting Information Figure S1.1** the PCs from the nine particles studied at t=0 h and extracted by Wire are shown. The Si substrate and CURC inside μPLs are respectively paired to the PC1 and PC2 while PC3 is not matched by the PLGA spectrum, as discuss in the main text of this paper. From Supplementary Fig.1a we notice an absorption-like behavior of PC1 depending to the high reduction of the Silicon signal in the spectra inside the particle respect the outside. In other words, when the CURC signal increase, the Si is reduced.

In the **Supporting Information Figure S1.2 (a)** and **(b)** respectively the PC1 (i.e. Si spectrum like) and PC2 (i.e. CURC spectrum like) profiles are shown. Here different colors are attributed to different spectral regions: *i)* Blue for Si peak; *ii)* Orange for the main CURC resonances; *iii)* Green for a "no-signaling" region. In Supplementary Fig.2 (c), the same regions are marked in the *scatter-plot* between PC1 and PC2 scores. Due to the orthogonality of the transformation induced by the PCA, the quasi-orthogonality between the Blue and the Orange masked points is expected. We suppose that the not perfect perpendicularity of these data is due to the baseline function that introduces a sort of correlation between them. From the scatter-plot is also possible to highlights the spectral region where not information's are stored (i.e. Green masked points). This is a useful tool to optimize the spectral window adopted for μRAMAN mapping reducing more that is possible the acquisition time.

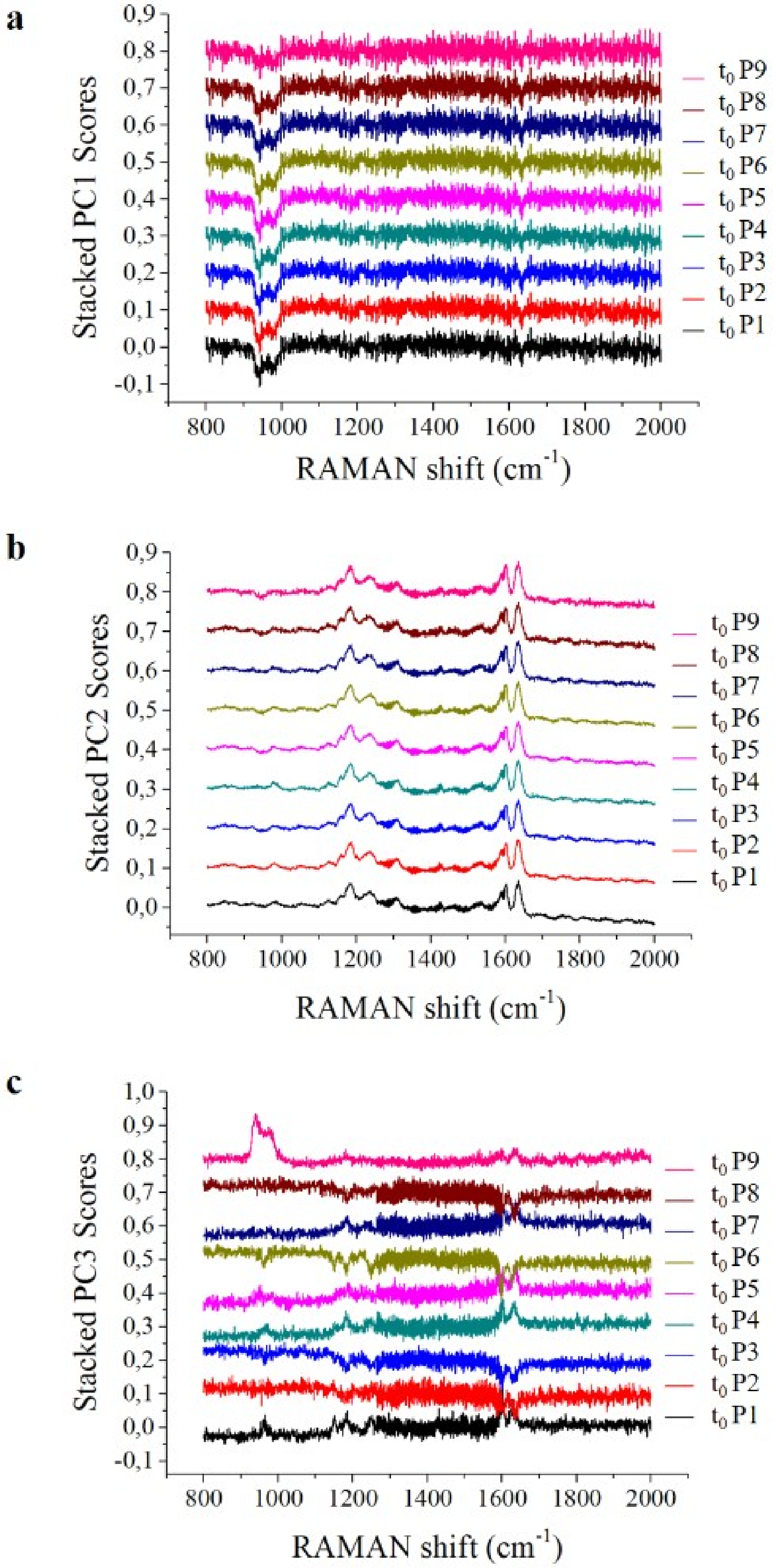
a
Stacked PC1 Scores
0,9
0,8
0,7
0,6
0,5
0,4
0,3
0,2
0,1
0,0
-0,1
800
1000
1200
1400
1600
1800
2000
RAMAN shift (cm^{-1})
t_0 P9
t_0 P8
t_0 P7
t_0 P6
t_0 P5
t_0 P4
t_0 P3
t_0 P2
t_0 P1
b
Stacked PC2 Scores
0,9
0,8
0,7
0,6
0,5
0,4
0,3
0,2
0,1
0,0
800
1000
1200
1400
1600
1800
2000
RAMAN shift (cm^{-1})
t_0 P9
t_0 P8
t_0 P7
t_0 P6
t_0 P5
t_0 P4
t_0 P3
t_0 P2
t_0 P1
c
Stacked PC3 Scores
1,0
0,9
0,8
0,7
0,6
0,5
0,4
0,3
0,2
0,1
0,0
-0,1
800
1000
1200
1400
1600
1800
2000
RAMAN shift (cm^{-1})
t_0 P9
t_0 P8
t_0 P7
t_0 P6
t_0 P5
t_0 P4
t_0 P3
t_0 P2
t_0 P1

Supporting Information Figure S1.2. PC1 (a), PC2 (b) and PC3 (c) principal components extracted from Raman spectra of nine samples at the initial time of release $t = 0$. Displayed loading profiles indicate how much individual frequencies contribute to a specific principal component.

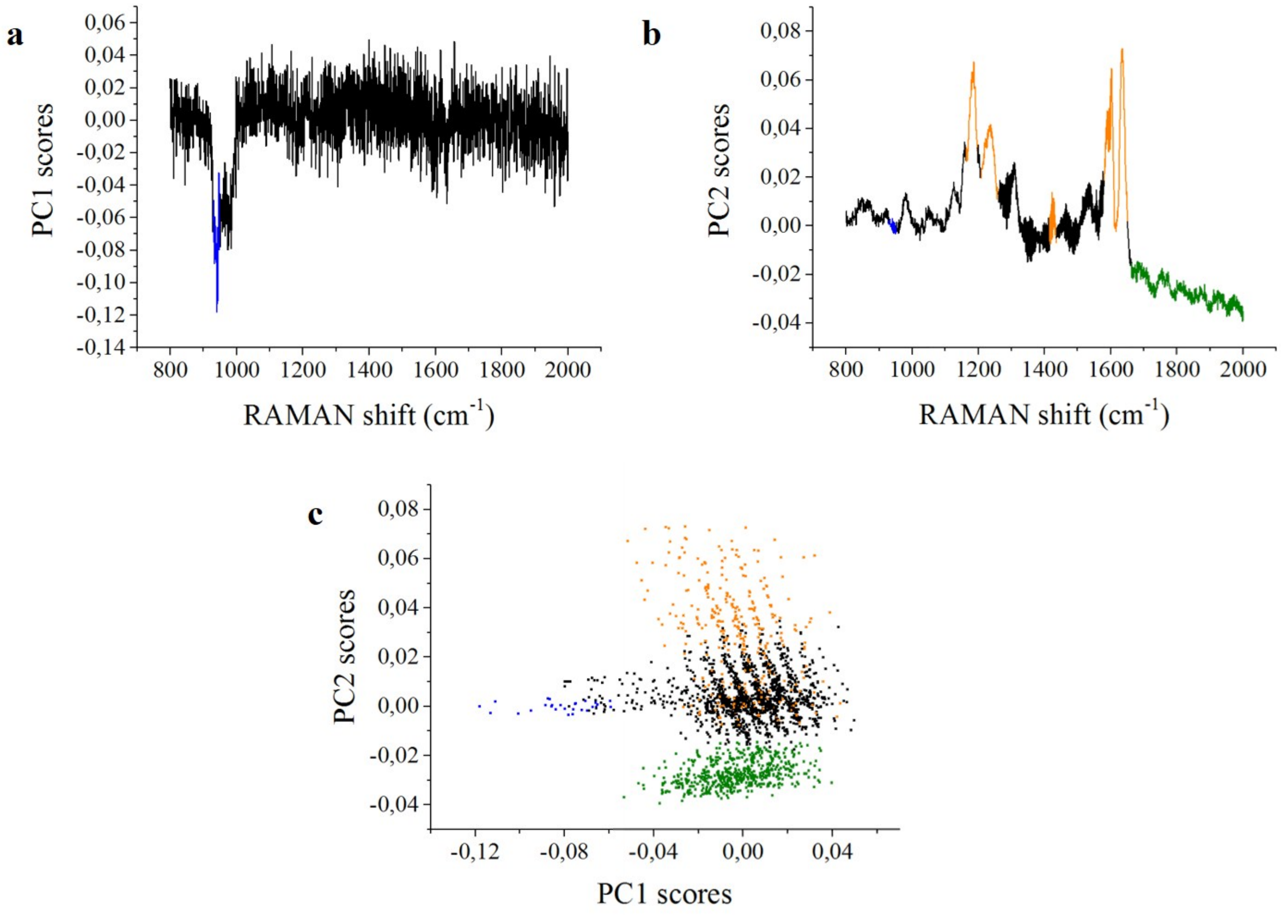

Supporting Information Figure S1.2. PC1 (a) and PC2 (b) loading curves associated to a micro-plate at the initial time of release. In the images, colours indicate different spectral regions: Silicon peak (blue), principal curcumin resonances (orange), no signal region (green). The PC1 vs PC2 scatter plot in (c) indicates that the components associated to Silicon (blue) and curcumin (orange) are orthogonal (mutually independent).

SI-2. Details on the micro Raman post processing - 1st Normalization. In this Supplementary section details on the first normalization (i.e. temporal normalization) on a reference Si sample and base-line subtraction are reported. In the **Supporting Information Figure S2.1a** the principal peak from Si at t=0 is shown and a *Voigt* fit is applied by Origin. Voigt function must be applied to RAMAN resonances due to its intrinsic mixed nature between a Gaussian and a Lorentzian shape. In the insert of the **Supporting Information Figure S2.1a** the information from the fit are reported. This fit is applied to Si reference for each time point. From this result, we acquired the x_c central frequency and the related intensity is used to calculate the 1st Normalization Constants as reported in the **Supporting Information Figure S2.1b**. How already explain in the main text of this paper, the x_c frequencies, indicated as K_{Si}, are not constant and an average value of 520.3±0.2 cm^{-1} is measured.

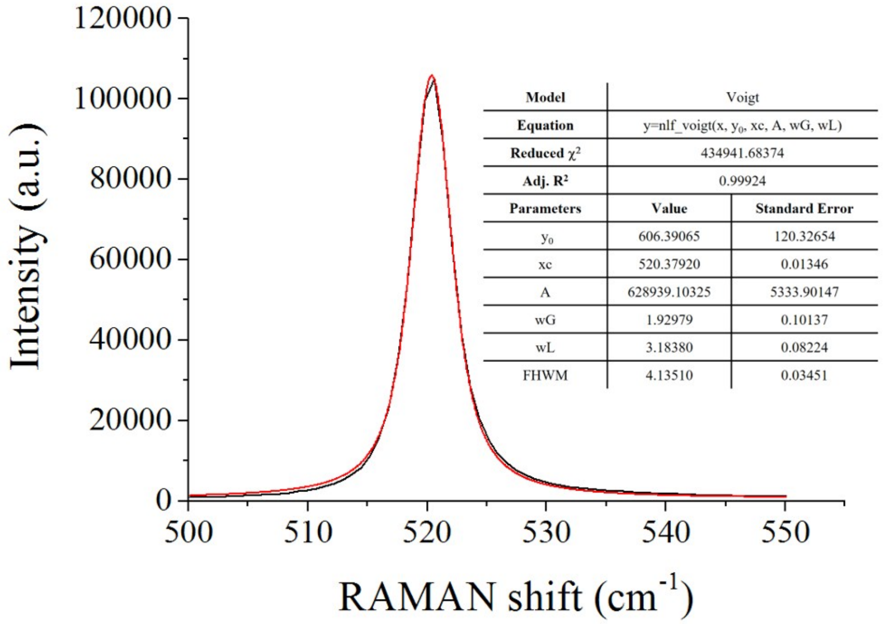

1st Normalization Constants (k_t)

k_0	7.11 x 10^{-4}
k_1	7.11 x 10^{-4}
k_2	7.11 x 10^{-4}
k_3	6.84 x 10^{-4}
k_4	6.43 x 10^{-4}
k_5	6.29 x 10^{-4}
k_6	5.95 x 10^{-4}
k_7	5.87 x 10^{-4}

a **b**

Supporting Information Figure S2.1. Silicon spectra measured on a reference sample are used for the first normalization (a). Spectra are fitted using a Voigt function. Table in the inset (b) reports the amount of variation of the constants k used for the first normalization at different time frames.

SI-3. Details on the micro RAMAN post processing – Baseline considerations. Definition and subtraction of baseline from spectra is a common issue in spectroscopy. The presence of a baseline superposed to the spectra can be considered as a "reality effect". This is due to the variation of experimental condition during the acquisition of a measure as temperature variation, laser fluctuation or someone that open the lab door. Clearly all of them are not predictable a priori but only after the spectrum acquisition. Several techniques are used to define a polynomial function (one for each spectrum) that can be attributed to the baseline and subtracted to the spectrum. All of them, more or less accurate, have the limitation that if is unknown the nature of the baseline there is no way to verify if the selected function is really correct. The risk is to subtract a different function altering the spectrum. This is one of the main reasons because; also if theoretically Raman spectroscopy is a quantitative analysis, up to know is used only for qualitative evaluations. To overcome this problem the scientific community had start to work on different experimental configurations based on the Raman effect (i.e. CARS, SERS, TERS, etc…). Differently, the innovative analytical approach presented in this paper, evaluate the baseline on single frequencies over the entire map. This changing in the point of view is crucial to reach the Micro-Raman quantification, as stressed in the main text. In the **Supporting Information Figure S3.1** the histograms of the Intensity distribution (after the 1st normalization) over a map at five fixed frequencies for t=0 h is shown. The choice of these frequencies and the histogram interpretation is already described in the main text. What we notice is that the Gaussian distribution of the data referred to the map-points outside the particle (lower Intensity peak) has different central position (i.e. central intensity) for each frequency. Due to the fixed frequencies of the analysis, theses distributions should be centred to zero-intensity (i.e. no signal where there is not curcumin). Moreover, the not linear behaviour in the background position is what we interpreted has a kind of map-baseline. In that way, we can define a punctual baseline that can be rigidly subtracted to the intensities of a specific frequency on the entire map, as described in section 5.7 of Materials and Methods.

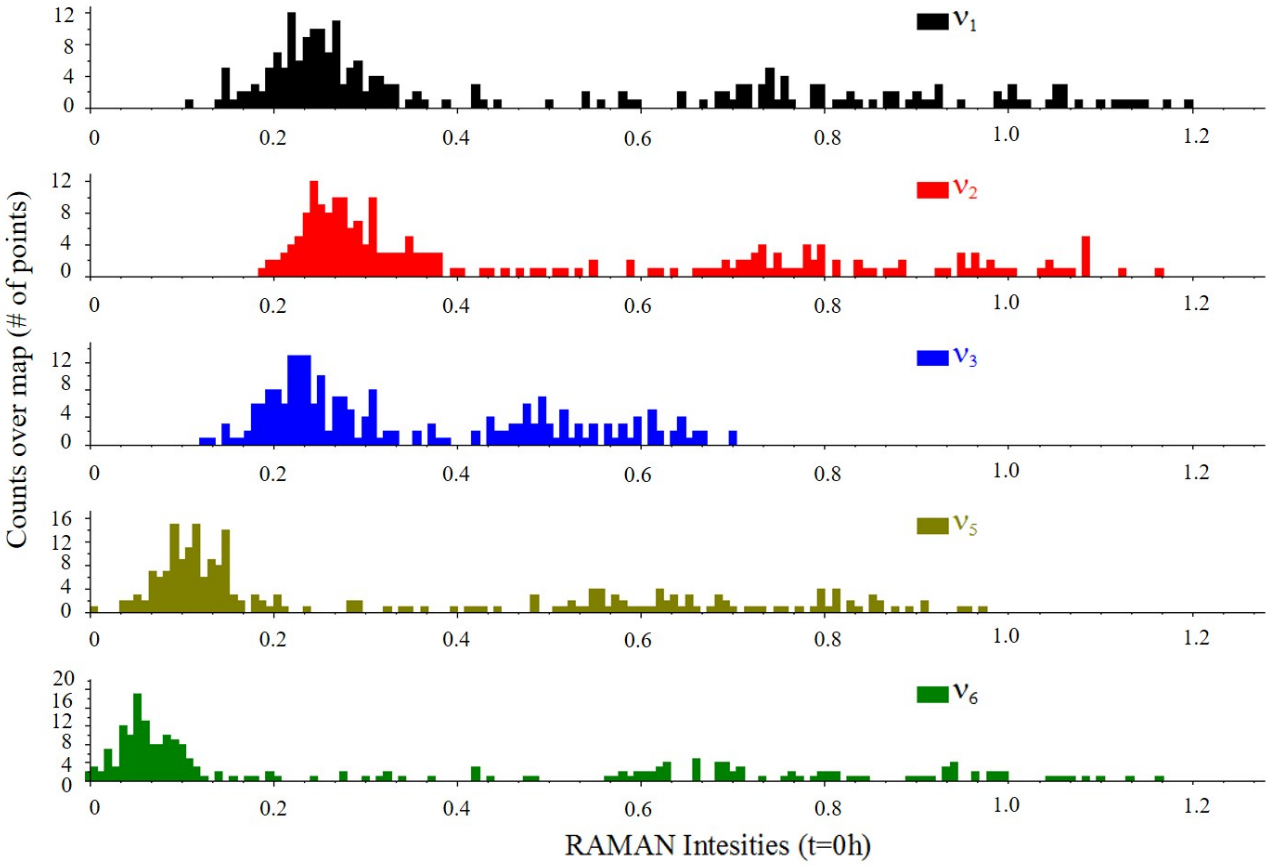

Supporting Information Figure S3.1. Raman intensity distributions obtained by the first normalization method at different central frequencies from ν_1 to ν_6 for $t = 0$.

SI-4. Evaluation of μPL bunch purity by Micro-Raman quantification. As already explain in the paper, one of the main advantages of a Micro-Raman analysis compared to the HPLC is the possibility to study individual particles instead of a bunch. Studies on the entire population at once will considers also whatever is present in the solution where particles are dispersed in and, depending on the process used to synthetize the particles, it can alter their real pharmacological characteristics, for example for the presence of debris. In the **Supporting Information Figure S4.1** we compared the CURC release evaluated by μRAMAN quantification with the HPLC performed on a "purified" (as shown in Fig. 5) and "not purified" bunch of particles. How expected, μRAMAN analysis can be considered as a reference for the purity of the sample from debris just because it is measured from the particles and no fragments are taken in account. From the **Supporting Information Figure S4.1** it is possible to understand how critical is this consideration on the release behaviour. In effect, to have a controlled release only by the engineering of the particles, the definition of a purity reference becomes a need.

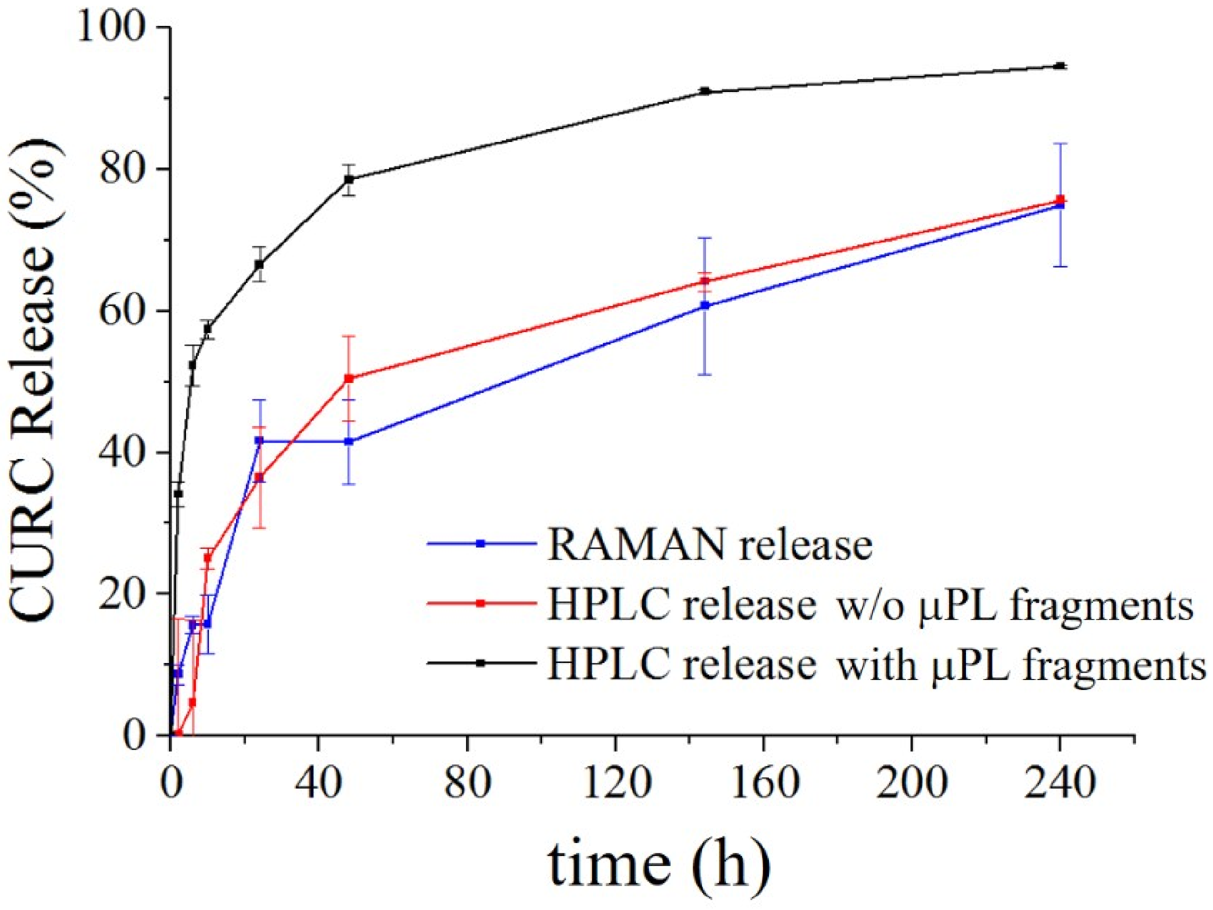

Supporting Information Figure S4.1. Comparison between curcumin release curves derived using (i) our Raman based method of analysis (blue); (ii) HPLC performed on a highly-purified sample (red); (iii) HPLC performed on an untreated sample (black).

SI-5. An alternative method base on clustering to separate the signal from the background in the micro plates. We performed unsupervised clustering of Raman spectra acquired on the entire region of interest setting the maximum number of clusters as 2. Then, we compared the positions in the plane of the spectra partitioned into two clusters to the position of the micro-platelet placed on the stage the micro Raman set up for analysis. From the comparison, it results that the clustering algorithm discriminated between in-silicon and out-of-silicon spectra with a precision as high as $\sim 80\%$, with the highest assignment errors at the border of the particle. While this level of accuracy is relevant, clustering alone does not achieve perfect matching between the spectra and their position on the sample surface. On the contrary, histogram description of spectral intensities associated to the $1630\ cm^{-1}$ Raman band enables direct graphical representation of data, reduces uncertainty, and achieves an efficiency of selectivity near unity.

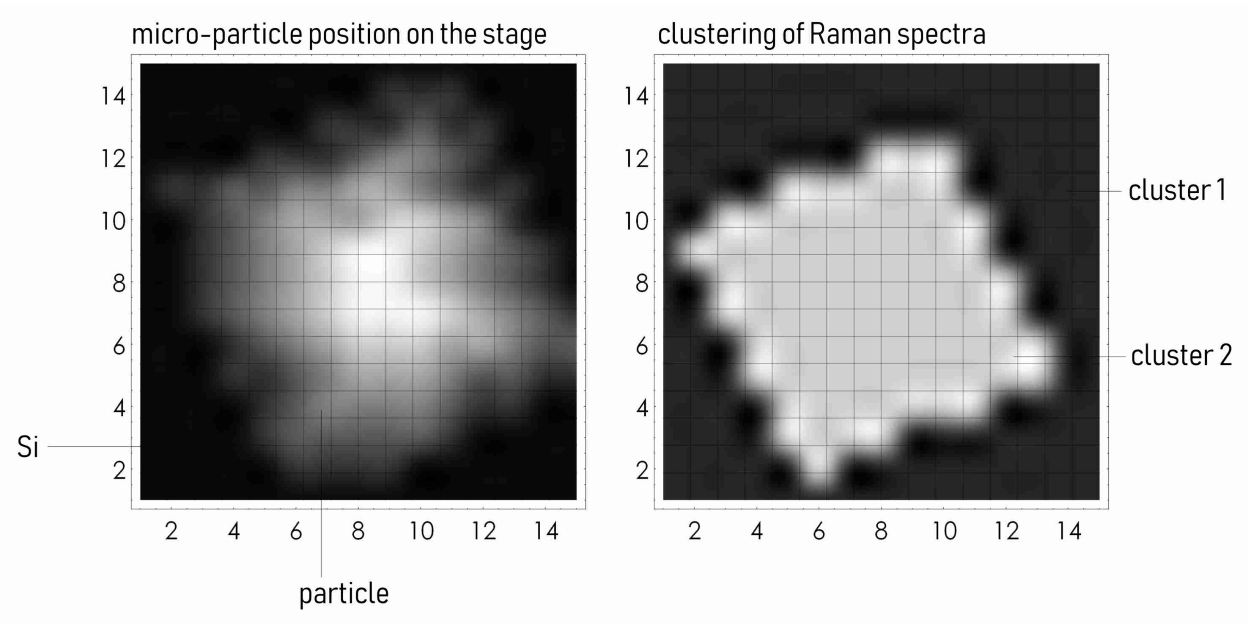

Supporting Information Figure S5.1

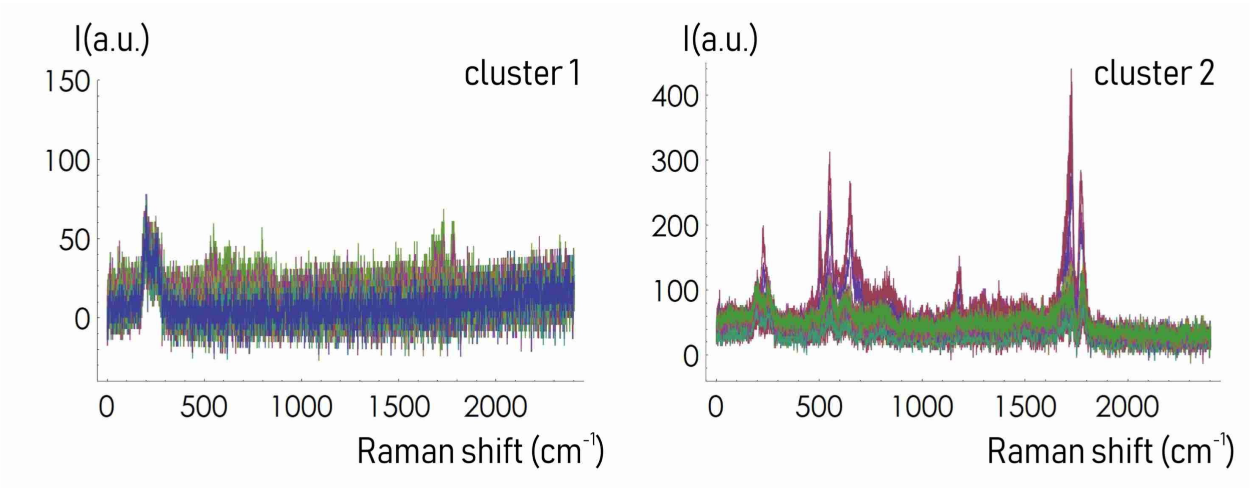

Supporting Information Figure S5.2

SI-6. An alternative method of analysis of Raman spectra, derivation of the drug release profile and comparison with the main findings of the paper. For each time step, for each particle per time step, we separate all spectra of a particle from the background using unsupervised clustering of Raman spectra setting the maximum number of clusters as 2. The clustering algorithm discriminates between in-silicon and out-of-silicon spectra. Then, we baseline correct all spectra of a particle using an automatic procedure. After correction, we find the average S of all the spectra associated to a particle. Then, we calculate the integral of the absolute values of S extended over the entire spectral range:

$$I_S = \int_{\nu_o}^{\nu_f} |S| d\nu \qquad \text{(S 6.1)}$$

We then further select from the pool of spectra all those spectra with a characteristic integral I that departs less than $0.5\ \sigma$ from I_S, i.e. it is within the band $I = I_S \pm 0.5\ \sigma$. This serves as a procedure to eliminate from the sample all anomalous spectra. Then, we take the average of the selection: the mean spectrum of a restricted group of spectra represents the characteristic spectrum of a particle, that will be used for deducing the release profile (**Supporting Information Figure S6.1**). Then, we report all means measured at different time frames in the same diagram (**Supporting Information Figure S6.2**). Of the means, we select a frequency band centered around $1630\ cm^{-1}$ (**Supporting Figure S6.2**), and calculate the release profile from the particle as $R(t) = 1 - I_t/I_o$, where I_t is the Raman spectrum intensity measured at $1630\ cm^{-1}$ at a specific time t, and I_o is the intensity at initial time. The fraction of drug released over time departs from that measured in the main article using the second normalization and HPLC methods to a great extent (**Supporting Information Figure S6.3**). Results reinforce the main findings of the work, that a second normalization of data based on the intensity of silicon band over the entire spectral range is necessary for a correct processing of data and for deriving the quantitative profile of drug release.

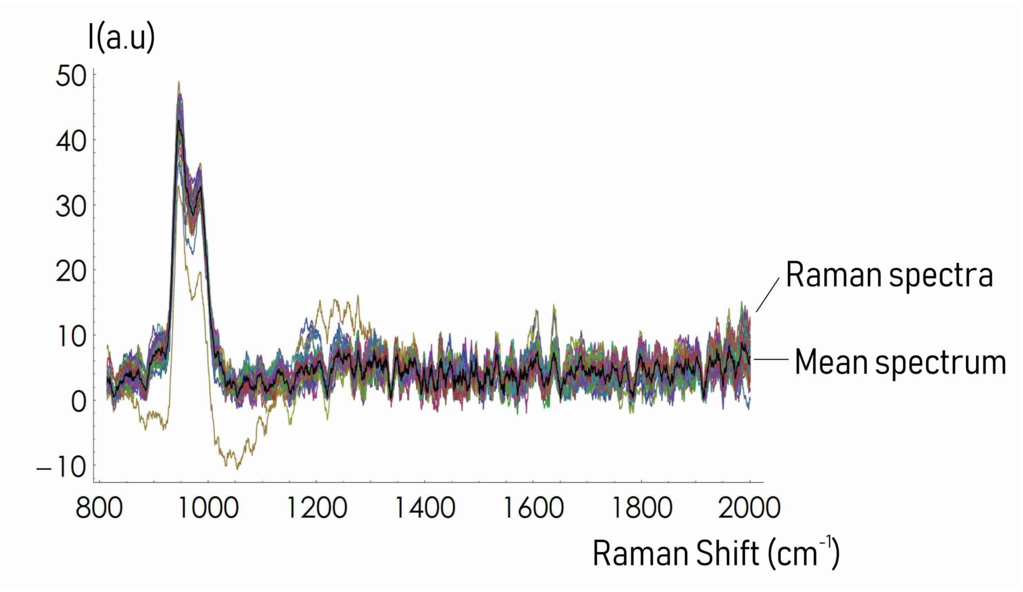

Supporting Information Figure S6.1

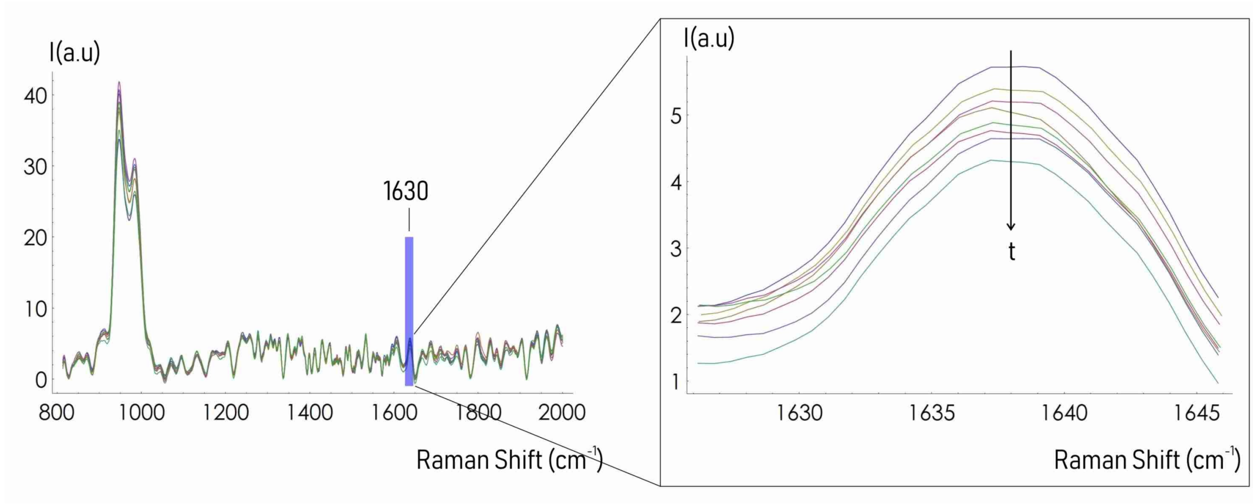

Supporting Information Figure S6.2

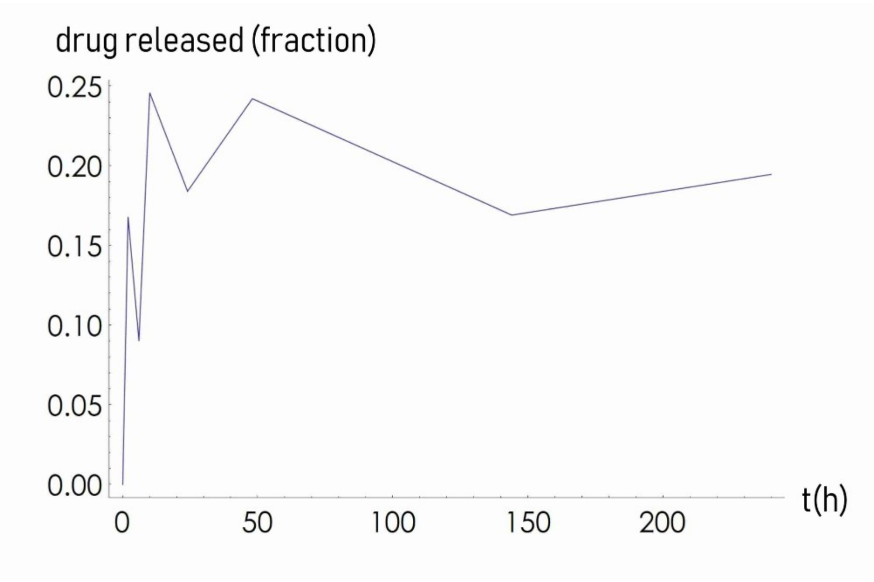

Supporting Information Figure S6.3

Automatic procedure for base line correction. **Supporting Information Figure S6.4.1** reports a raw Raman spectrum before correction. Convolution with a uniform array of ones allows to smooth data as in **Supporting Information Figure S6.4.2**. Then, we choose an arbitrary number of intervals $p = 10$ into which the frequency range is divided. Per each interval, we find the point of the spectrum with minimum intensity as in **Supporting Information Figure S6.4.3**. In doing so, we find p interpolation points that are not necessarily equally spaced in the considered frequency range (we shall call this set of points, P). Then, we use an interpolation function to interpolate among the points P in the spectrum (**Supporting Information Figure S6.4.4**) and find a baseline for the spectrum. Interpolation works by fitting polynomial curves between successive data points. The degree of the polynomial curves is here fixed as $n = 2$. Upon subtraction of the baseline to the original spectrum, we derive the baseline corrected spectrum (**Supporting Information Figure S6.5**).

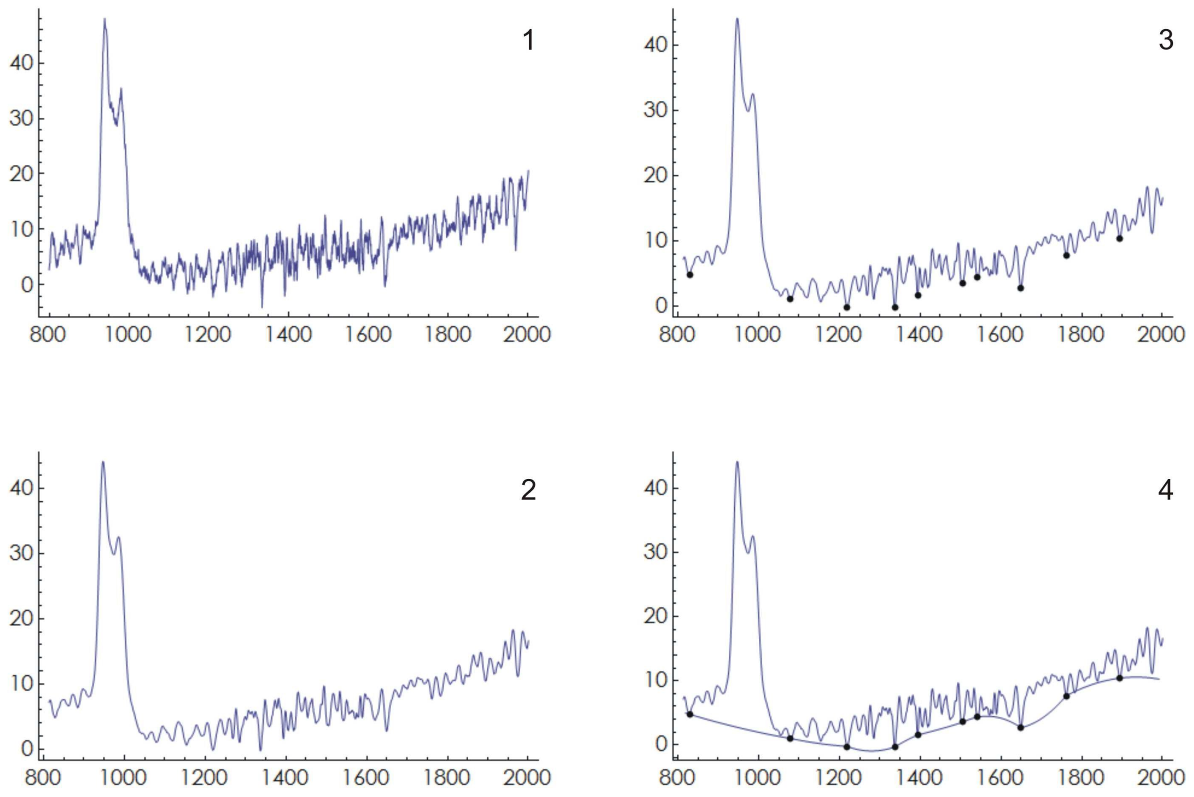

Supporting Information Figure S1.4

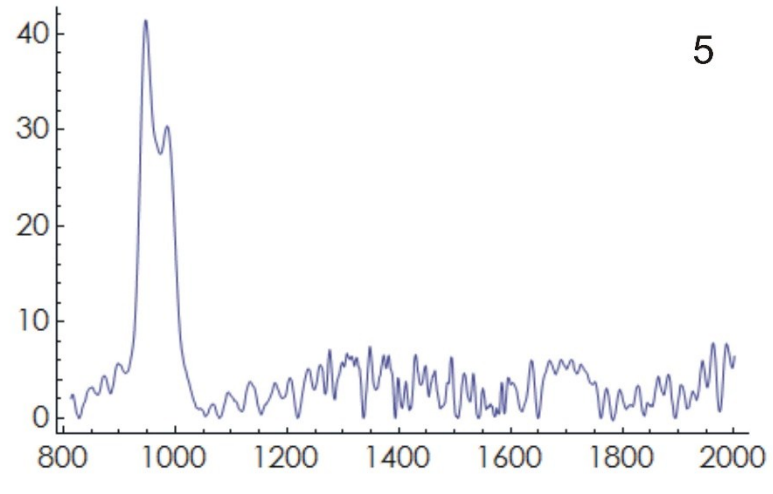

Supporting Information Figure S1.5
